# Post-traumatic growth and influencing factors of parents with children with Duchenne muscular dystrophy: a cross-sectional survey study

**DOI:** 10.1186/s13052-025-01840-z

**Published:** 2025-02-21

**Authors:** Li Xu, Meili Liu, Yuewei Chen, Liwen Wu, Siyi Gan, Jianhui Xie, Jos M. Latour

**Affiliations:** 1 Department of Neurology, Hunan Children’s Hospital, Changsha, China; 2 Nursing Department, Hunan Children’s Hospital, Changsha, China; 3 School of Nursing and Midwifery, Faculty of Health, University of Plymouth, Plymouth, UK; 4 Curtin School of Nursing, Curtin University, Perth, Australia

**Keywords:** Duchenne muscular dystrophy, Post-traumatic growth, Social support, Care burden, Parents, Children, Family

## Abstract

**Background:**

The aim of the study was to identify the post-traumatic growth status and influencing factors of parents with children with Duchenne muscular dystrophy (DMD).

**Methods:**

We adopted a cross-section survey study. Between February and December 2022, 181 parents responded to the survey including a participants’ characteristics section, post-traumatic growth assessment scale, caregiver burden scale, and social support assessment scale. Multiple linear regression analysis was used to investigate influencing factors of post-traumatic growth.

**Results:**

The mean score of post-traumatic growth of parents was 56.66 (SD ± 18.67). Post-traumatic growth was positively correlated with social support (*r* = 0.452, *P* < 0.01) and negatively correlated with care burden (*r* = -0.207, *P* < 0.01). Multiple linear regression showed that the child’s age, course of disease, self-care ability, parent’s working condition, residence, education, number of children, and health status were the main influencing factors for the post-traumatic growth of parents (*P* < 0.001).

**Conclusion:**

The post-traumatic growth of parents with children with DMD was at a moderate level. Healthcare professionals should pay attention to the psychological state of parents with children with this rare disease and promote post-traumatic growth through psychological mindfulness interventions, strengthening family and social support, and providing care knowledge and skills.

## Background

Duchenne muscular dystrophy (DMD) is a severe X-linked recessive degenerative neuromuscular disease. It is an idiopathic genetic disease in male children, with an incidence between 1/3500 and 1/5000 in male live births [1–3]. Most children experience rapid disease progression after onset, and generally have no special manifestations before the age of 1 year. Only a small number of patients may experience delayed motor development or landing on the toes [4]. Generally, most children show typical symptoms between the ages of 2 and 5 years [5], such as walking difficulties, duck gait, difficulties in climbing stairs or standing up from the prone position.

Due to the severity of the disease itself, family members and carers might experience a certain degree of care burden. The burden can be influenced by insufficient social support systems over time. The negative psychological experiences might result in anxiety and depression leading to traumatic events and consequently increasing the psychological pressure of family life of children with DMD [6].

Post-traumatic growth refers to ‘the positive psychological, cognitive, and emotional changes experienced by individuals in the process of active struggle after encountering extremely challenging crises, traumatic life events or situations’ [7]. Studies have pointed out that post-traumatic growth may affect stress levels of patients with a disease, improve the self-efficacy and quality-of-life, and provide positive guidance for the psychological intervention [8, 9]. At present, research related to post-traumatic growth in patients and family members mainly focuses in areas such as oncology, intensive care, and functional impairment [10–12]. There remains a gap in the evidence base of post-traumatic growth of parents with children with DMD. Therefore, the aim of our study was to identify the post-traumatic growth status and to analyze influencing factors of parents with children with Duchenne muscular dystrophy.

## Methods

### Design and ethics

The study adopted a cross section survey design. The study protocol was approved by the Research Ethics Committee of Hunan Children’s Hospital with the study number: HCHLL-2022-30. The reporting guideline ‘The Strengthening the Reporting of Observational Studies in Epidemiology (STROBE) Statement: guidelines for reporting observational studies’ has been used to report this study [13].

### Setting

This study was conducted at the neurology outpatient department of Hunan Children’s Hospital, involving 181 families of pediatric patients with diagnosed with DMD. According to the 2014 edition of Medical Statistics, the sample size of a multi-factor survey study should include 10–20 responses per factor variable [14]. Our study involved a total of 15 influencing factor variables. Therefore, the sample size was estimated between 150 and 300 cases. The final sample size determined in our study was within this range.

### Participants

From February to December 2022, a convenience sampling technique was used to conduct the survey among 181 family members of children with DMD in our neurology outpatient department. Inclusion criteria were: (1) The patient must meet the diagnostic criteria of DMD, namely progressive proximal limb muscle weakness, gastrocnemius muscle pseudohypertrophy, positive Gowers sign, elevated serum creatine kinase, and myogenic damage in electromyography; (2) The patient must have completed DMD gene detection; and (3) The parents or carer of the patient voluntarily participated in this study and signed an informed consent form. Exclusion criterium was: 1) parents were not able to read Chinese.

### Data sources and measurements

#### Participants’ characteristics

The research team designed the participants’ characteristics variable based on the literature and group discussions defined the demographic characteristics of children and parents, including age, gender, course of disease, and self-care ability, whether they were a non-single child, their parents’ education level, health condition, the parents’ relationship with the children, the family’s annual income, and their culture. These questionnaires were designed by the research team based on literature review and group discussions to assess aspects such as self-care ability, which involves evaluating whether children can complete daily life activities (eating, dressing, bathing, going up and down stairs, etc.). The evaluation of health condition is based on the self-evaluation of the research subjects: poor (combining with serious illnesses, chronic illnesses, physical disabilities, or without a clear diagnosis of the disease, self perceived poor physical condition ); normal (normal functioning of an individual’s major systems and organs, absence of diseases, physical condition, and physical strength, all of which belong to the category of normal individuals); in good shape (self perceived good physical condition). Settlement investigation involves evaluating the living areas of the research subjects, which are categorized into rural, city, and suburb areas.

#### Post-traumatic growth inventory

The post-traumatic growth inventory is a 20-item scale used to assess the degree of positive change after trauma [15] and includes five dimensions, namely: relationship with others (3 items), new possibilities (4 items), personal strength (3 items), self-transformation (4 items), and philosophy of life (6 items). Scores range from 0 to 5, with a total score of 0 to 100. The Cronbach’s α coefficient of the scale was 0.874, and the Cronbach’s α coefficient of each dimension was 0.611 to 0.796, which had high reliability and validity and could be used to evaluate the positive changes in individuals after experiencing trauma [15].

#### Zarit caregiver burden interview (ZBI)

The Zarit caregiver burden interview (ZBI) scale, developed by Zarit et al. [16], can effectively measure caregiver burden and has been widely used. The scale consists of 22 items, each of which represents caregiver burden using a 5-point scale ranging from 0 to 4, with a total score ranging from 0 to 88. The higher the score, the heavier the caregiver burden. The burden is graded as follows: no or very mild burden is 0 to 19 points, mild burden is 20 to 39 points, moderate burden is 40 to 59 points, and severe burden is more than 60 points. The scale has good reliability and validity, and the total Cronbach’s α coefficient is 0.87 [16].

#### Social Support rating scale

The Social Support Rating Scale (SSRS) scale was designed by Xiao Shuiyuan [17] and is mainly used to measure the level of social support of individuals. The scale contains 10 items, and each item has different options and scores, with a total score of 66 points. The higher the score, the better the individual’s level of social support. The scale is widely used in China and has good reliability and validity. The test–retest reliability is 0.92, and the consistency coefficient of each item is between 0.89 and 0.94 [17].

### Data collection

An electronic online questionnaire (questionnaire star https://ks.wjx.top/vm/YjhqxOA.aspx# ) was used. Before the survey, researchers invited eligible study participants, provided written and verbal information of the study, and ensured that all participants had voluntarily enrolled in the study, had carefully read the study information, and signed the consent form. The main purpose, content, and privacy protection statements of the questionnaire were stated on the homepage of the survey. An electronic informed consent form was signed by the participants. Each IP address of the questionnaire could only be filled in once, and the time to complete the survey was around 30 min. All answer options needed to be completed before submission to ensure the effective recovery rate of the questionnaire.

### Statistical analysis

The statistical software IBM-SPSS version 25.0 was used to analyze the data. Statistical description was carried out according to the type of data. Measurement data conforming to normal distribution were expressed as mean and Standard Deviation (SD), and count data were expressed as frequency and percentage. One-way analysis of variance was used to analyze the factors affecting the post-traumatic growth of parents of children with DMD, Pearson’s correlation test was used to analyze the correlation of post-traumatic growth, and multiple linear regression was used to analyze the multiple factors of post-traumatic growth. A value of *P* < 0.05 was considered statistically significant.

## Results

A total of 191 questionnaires were distributed to parents and 181 valid questionnaires were finally collected, with an response rate of 94.76%.

### Study population

The mean age of children with DMD was 9.41 years, the course of disease in mean years was 4.82 years, and the mean age of parents in years was 38.59 (Table 1.)

**Table 1:** Characteristics of study participants and single-factor analysis of post traumatic

Item	Case (%)	Post-traumatic growth Score	Test Statistic	*P* value
Child’s age			14.896	<0.001
≤3 years old 4–6 years old >6 years old	6 (3.3) 36(19.9) 139(76.8)	30.33 ± 15.90 47.39 ± 18.85 60.20 ± 17.03		
Course of disease			16.874	<0.001
≤1 years old 2–5 years old >5 years old	28(15.4) 103(57.0) 50(27.6)	39.57 ± 22.67 58.78 ± 16.94 61.88 ± 13.99		
Self-care ability			32.143	<0.001
Takes care of oneself Assisted Relies entirely on others	62 (34.3) 101 (55.8) 18 (9.9)	69.12 ± 12.80 51.92 ± 16.21 40.39 ± 24.02		
Parent’s age			0.476	0.622
≤35 years old 36–40 years old >40 years old	62(34.2) 66(36.5) 53(29.3)	58.42 ± 17.86 56.29 ± 18.53 55.08 ± 19.92		
Gender			2.169	0.143
Male Female	38(21.0) 143(79.0)	52.71 ± 16.60 57.71 ± 19.10		
Medical expenses			0.210	0.811
Free National health insurance At one’s own expense	1(0.6) 8(4.4) 172(95.0)	47 59.25 ± 22.82 56.60 ± 18.67		
Marital status			0.008	0.928
Married Single	172(95.0) 9(5.0)	56.69 ± 18.93 56.11 ± 13.66		
Working condition			4.383	0.038
Working Unemployed	87(48.1) 94(51.9)	59.656 ± 18.39 53.89 ± 18.60		
Family income annually			1.650	0.180
CNY ≤ 50,000 CNY 50,000-100,000 CNY 100,000-150,000 CNY >150,000	105(58.0) 54(29.8) 19(10.5) 3(1.7)	54.52 ± 17.86 58.06 ± 20.73 62.58 ± 16.44 69.00 ± 10.54		
Settlement			4.842	0.009
Rural area City Suburb	95(52.5) 54(29.8) 32(17.7)	52.80 ± 18.30 61.94 ± 17.15 58.00 ± 21.26		
Educational background			4.212	0.007
Primary school Middle school University Master’s degree or above	23(12.7) 90(49.7) 65(35.9) 3(1.7)	45.78 ± 15.00 56.00 ± 18.76 61.15 ± 18.63 62.67 ± 5.69		
Number of children			20.391	<0.001
1 2 ≥3	57(31.5) 99(54.7) 25(13.8)	65.47 ± 18.50 55.88 ± 15.56 39.68 ± 18.43		
Health condition			37.824	<0.001
Poor Normal In good shape	18(9.9) 104(57.5) 59(32.6)	31.33 ± 16.43 54.93 ± 15.19 67.44 ± 16.44		

CNY = Chinese yuan renminbi

### Single-factor analysis of post-traumatic growth

The results of the single-factor analysis of post-traumatic growth of parents with children with DMD showed that the age of the children, course of disease, self-care ability, working status, residence, education level, number of children, and health status of parents were statistically significant (*P* < 0.05), as presented in Table 1.

### Correlation analysis of post-traumatic growth, care burden, and social support

The scores of each dimension of the post-traumatic growth of parents are presented in Table 2. The care burden of parents with children with DMD was negatively correlated with social support (*r* = -0.230, *P* < 0.01), and post-traumatic growth was positively correlated with social support (*r* = 0.452, *P* < 0.01). Post-traumatic growth was negatively correlated with care burden (*r* = -0.207, *P* < 0.01) (Table 3).

**Table 2:** Post-traumatic growth, care burden, and social support (*n* = 181)

Dimension	Score (score, x ± s)
Personal strength	9.15 ± 3.73
New possibilities	8.49 ± 4.81
Philosophy of life	20.82 ± 5.65
Relationship with others	7.07 ± 3.55
Self-transformation	11.13 ± 4.56
Total score post-traumatic growth	56.66 ± 18.67
Care burden	45.02 ± 18.22
Social support	35.66 ± 7.47

**Table 3:** Correlation analysis of post-traumatic growth with care burden and social support

	Social support	Care burden	Post-traumatic growth
Social support	1		
Care burden	− 0.230**	1	
Post-traumatic growth	0.452**	− 0.207**	1
mean	35.66	45.02	56.66
SD	7.46	18.22	18.67

^**^ Represents 0.01 (double tail) with significant correlation. SD = Standard Deviation

### Multiple linear regression analysis of post-traumatic growth

Using the post-traumatic growth of parents with children with DMD as the dependent variable and meaningful variables from univariate analysis as independent variables, multiple linear regression analysis was conducted, and the stepwise regression method was used to screen the independent variables (α In = 0.05, α Out = 0.15). The assignment of independent variables is presented in Table 4. The results showed that seven variables, namely child’s age, course of disease, self-care ability, parents’ gender, education level, health status, and number of children, were included in the regression equation explaining 53.0% of the total variation (Table 5).

**Table 4:** The assignments of independent variables

Argument	Assignment
Age of the child (years)	1=<3; 2 = 4 ∼ 6; 3>6
Course of the disease (years)	1=<1; 2 = 2 ∼ 5; 3>5
Self-care ability	1 = takes care of oneself; 2 = assisted; 3 = relies entirely on others
Parents’ age (years)	1=<35; 2 = 36 ∼ 40; 3>40
Gender	1 = male; 2 = female
Medical expense	1 = free; 2 = national health insurance; 3 = at one’s own expense
Marital status	1 = married; 2 = single
Working condition	1 = working; 2 = unemployed
Family income annually (CNY)	1 = CN<≤50,000; 2 = CNY 50,000-100,000; 3 = CNY 100,000-150,000; 4 = CNY>150,000
Settlement	1 = rural area; 2 = city; 3 = suburb
Educational background	1 = primary school; 2 = middle school; 3 = university; 4 = Master’s degree or above
Number of children	1 = 1; 2 = 2; 3=>3
Health condition	1 = poor; 2 = normal; 3 = in good shape

CNY = Chinese yuan renminbi

**Table 5:** Multiple linear regression analysis of factors influencing post traumatic growth

	Standard error	Standardization coefficient	t	*p*-value	95.0% CI
(Constant)	10.427	-	2.749	0.007	(8.083, 49.245)
Parents’ health status	1.871	0.234	3.81	<0.001	(3.436, 10.823)
Child’s self-care ability	1.638	-0.346	-6.36	<0.001	(-13.647, 7.183)
Number of children	1.599	-0.232	-4.153	<0.001	(-9.799, 3.486)
Disease course of children	1.675	0.129	2.224	0.027	(0.419, 7.032)
Gender of Parents	2.37	0.137	2.642	0.009	(1.584, 10.938)
Children’s age	2.157	0.161	2.715	0.007	(1.6, 10.113)
Parents’ degree	1.448	0.122	2.259	0.025	(0.412, 6.128)

Note: *R* = 0.741, R2 = 0.549, adjusted R2 = 0.530, F = 5.102, *P* = 0.025. CI = Confidence Interval

## Discussion

Children disease experience can significantly impact on parental mental health leading to a wide spectrum of conditions ranging from unspecified chronic distress to specific nosological entity such as post-traumatic stress disorder (PTSD), anxiety, and depression [18, 19]. Nonetheless different studies report the possibility of a positive post-trauma experience called “post-traumatic growth” [8, 20, 21]. The aim of our study was to explore the post-traumatic growth status of parents with children with Duchenne muscular dystrophy and to identify influencing factors of post-traumatic growth. The post-traumatic growth of parents with children with DMD appeared to be on a moderate level and was positively correlated with social support and negatively correlated with care burden. The multiple linear regression identified that the age of the child, course of disease, self-care ability, parents’ working condition, residence, education, number of children, and health status were the main influencing factors for post-traumatic growth of parents.

The clinical manifestations of Duchenne muscular dystrophy (DMD) are progressive muscle atrophy and weakness, with 90% of patients experiencing pseudohypertrophy of the gastrocnemius muscle, most with myocardial damage, and a few with intellectual disabilities. The average survival rate is between 2 and 30 years [22]. As the disease progresses, families with DMD patients face a series of psychological and nursing challenges [23]. This causes serious trauma to the families of the affected children. Research has shown that a positive attitude towards the future can help injured individuals better cope with current traumatic events and improve the negative physical and mental experiences they bring [24]. Individuals who have experienced trauma and having post-traumatic growth are more confident and believe in their ability to solve difficulties and adapt to life. Our results are not only much lower than the post-traumatic growth levels of parents with children with cancer, but also lower than the post-traumatic growth levels of parents of children with autism [25–27]. This may be related to people’s better understanding of cancer than DMD, and the social support system and medical security systems in different countries are more complete than those in China. Post-traumatic growth is a process that requires a lot of time, energy, and inner motivation. In this study, 9.9% of the children were completely dependent on others, 15.5% of the children’s illnesses occurred within one year, and 3% of the children were less than three years old. The young age of children, poor self-care ability, worse health condition lead to heavy care burden for parents, which negatively impact on post-traumatic growth. Relevant studies have also shown that with the extension of the disease course, parents have more understanding of relevant knowledge, and their care ability is improved in the long-term care process [28]. Our study is also demonstrated that parents gain more post-traumatic growth along with disease course of children. Therefor, we should pay more attention to parents physical and mental health, provide corresponding support to achieve growth in long-term care.

In addition, our research results indicate that parents who reside in rural areas are in a state of resignation, have low levels of education, and have multiple children also have significantly lower post-traumatic growth scores than the average. This is consistent with the results of other studies [28, 29] and may be related to the increased burden of parents’ care, limited economic level, and susceptibility to physical and mental fatigue. In addition, due to the low level of education and limited resources available to parents living in rural areas, it affects their correct understanding and psychological response to the disease.We find remarkable differences in the level of post-traumatic growth among parents of different genders. Women are more capable of benefiting from complex life experiences [30].

Our study found that post-traumatic growth is positively correlated with social support, meaning that the higher the social support level of parents, the higher their post-traumatic growth level. Rare disease groups are increasingly receiving social attention. China Alliance for Rare Diseases issued “China Rare Disease Action Initiative 2030” in 2023. It advocated for multi-party collaboration and improve the social-care system [31]. Studies have shown that families with social support can better cope with the occurrence of stressful events [32], actively face the pain caused by the disease in their families lives and alleviate the negative impact of stressors on their families [33]. Strengthening social connections through social networks can alleviate parents’ physical and mental stress, improve their social adaptability, and enhance their mental health level [34]. Therefore, we believe that improving social support is an important factor in achieving post-traumatic growth. Healthcare professionals should provide social support to parents of children with DMD through various channels to raise the public awareness of rare diseases. Non-governmental organization (a charity, association, the Red Cross, etc) will play a certain role in providing social support and welfare for families.

The results of our study show that the higher the parents’ care burden, the lower the level of post-traumatic growth. Additionally, in our study, the care burden of parents was significantly higher than those with diseases such as cancer and autism [35, 36]. This may be related to the high mortality of DMD, the uncertainty of treatment, and the anxiety brought about by the upcoming long-term care pressure. A study indicated that primary caregivers of children are more likely to independently take on the main care tasks, making them more susceptible to physical and mental exhaustion [37]. If combined with economic pressure, they face greater pressure, leading to low levels of post-traumatic growth. This requires clinical healthcare workers to provide parents with more nursing knowledge and skills and pay attention to their psychological state to improve their care ability, reduce their care burden, and promote their post-traumatic growth. Meanwhile, our study found a negative correlation between care burden and social support as well, which is consistent with other studies [38]. Family is an important support system, and we should encourage parents and other family members to participate in the care process of children. Medical staff should also provide psychological and social support to parents to improve their physical and psychological stress caused by adverse nursing experiences.

Our study has some limitations to address. Firstly, although the parents surveyed in our study came from various parts of China, they completed the survey while visiting our hospital which could have influenced the completion of the self-administered online survey. Secondly, the study investigated the post-traumatic growth only at one time-point; the research team have not conducted a comparative analysis of the post-traumatic growth levels of parents at different stages of the patient’s illness, which is recommended to gain a full understanding of factors influencing post-traumatic growth in this cohort of family and carers with children with DMD.

## Conclusions

Our research results indicate that parents with children with DMD experience a moderate level of post-traumatic growth. The main influencing factors include the child’s age, course of illness, self-care ability, parents’ gender, educational background, health status, number of children raised, care burden, and social support. There is no effective support care plan for carers with patients with DMD. However, continuous interventions delivered by a multidisciplinary management such as early rehabilitation, drug treatment, and bone health management, the quality-of-life can be improved. Our medical and nursing staff have actively taken measures such as mindfulness intervention, family social support, and providing more care knowledge and skills to promote the post-traumatic growth of parents with children with DMD. Finally, healthcare managers and government officials should increase their attention to the patients and family with rare diseases and promote regional and national support to these patients and their family and friends.

## Data Availability

The datasets used and/or analysed during the current study are available from the corresponding author on reasonable request.

## Abbreviations

CNY: Chinese yuan renminbi
CI: Confidence Interval
DMD: Duchenne muscular dystrophy
SD: Standard Deviation
SSRS: Social Support Rating Scale
STOBE: Strengthening the Reporting of Observational Studies in Epidemiology
ZBI: Zarit caregiver burden interview
